# Developmental Defects in Trisomy 21 and Mouse Models

**DOI:** 10.1100/tsw.2006.322

**Published:** 2006-09-19

**Authors:** Jean Maurice Delabar, Revital Aflalo-Rattenbac, Nicole Créau

**Affiliations:** EA3508, Université Denis Diderot — Paris 7, 2 place Jussieu 75251 Paris Cedex 05, France

**Keywords:** Down syndrome, mouse model, trisomy 21, heart, brain, morphogenesis

## Abstract

Aneuploidies have diverse phenotypic consequences, ranging from mental retardation and developmental abnormalities to susceptibility to common phenotypes and various neoplasms. This review focuses on the developmental defects of murine models of a prototype human aneuploidy: trisomy 21 (Down syndrome, DS, T21). Murine models are clearly the best tool for dissecting the phenotypic consequences of imbalances that affect single genes or chromosome segments. Embryos can be studied freely in mice, making murine models particularly useful for the characterization of developmental abnormalities. This review describes the main phenotypic alterations occurring during the development of patients with T21 and the developmental abnormalities observed in mouse models, and investigates phenotypes common to both species.

### INTRODUCTION

Aneuploidies — copy number disorders of functional genomic elements — are common genomic disorders with a profound impact on the health of human populations. They have diverse phenotypic consequences, ranging from mental retardation and developmental abnormalities to susceptibility to common phenotypes and various neoplasms. This review focuses on the developmental defects of murine models of a prototype human aneuploidy: trisomy 21 (Down syndrome, DS, T21). The genetic cause of Down syndrome was identified 50 years ago, but the mechanisms by which these gene dosage errors induce phenotypic abnormalities remain unknown. The cause of mental retardation is also an open question, and it remains to be determined whether this abnormality results from changes in brain development, changes in synaptic plasticity and brain metabolism, or both. Murine models are clearly the best tool for dissecting the phenotypic consequences of imbalances that affect single genes or chromosome segments. Embryos can be studied freely in mice, making murine models particularly useful for the characterization of developmental abnormalities. This review describes the main phenotypic modifications occurring during the development of patients with T21 and the developmental abnormalities observed in mouse models, and investigates phenotypes common to both species.

### DEVELOPMENTAL ABNORMALITIES ASSOCIATED WITH TRISOMY 21

T21 is the most frequent type of aneuploidy (1/700 births and 500,000 patients in Europe). The DS phenotype was first described in 1846 by Seguin[1], and in 1866 by Down[2]. DS is caused by the presence of an extra copy of HSA21, as shown by Lejeune et al. in 1959[3]. T21 is frequently associated with mental retardation, congenital heart defects (mainly atrioventricular septal defect), abnormalities of the gastrointestinal tract, abnormalities of neuromuscular tone, atlanto-axial instability, characteristic facial and physical features, a high incidence of seizures, modified audiovestibular and visual functions, and early-onset Alzheimer's disease (AD). Only two of these features are observed in all DS patients: mental retardation and neuropathological modifications similar to those observed in the brains of AD patients (in DS patients over the age of 35 years). The best characterized and the most frequent developmental abnormalities concern the brain, the craniofacial and skeletal tissues, the heart and the gastrointestinal tract (Tables 1 and 2). Hypotonia, very frequently observed in neonates[4,5], is difficult to associate with a well-characterized developmental anomaly.

#### Brain Alterations

T21 strongly decreases intellectual quotient (IQ). Unlike children with normal development, children with DS display a progressive decline in IQ, beginning in the first year of life. The ratio of mental age to chronological age is not constant. By adulthood, IQ is usually in the moderately to severely retarded level (IQ 25–55), with an upper limit on mental age of approximately 7–8 years, although a few individuals have IQ in the lower normal range (70–80). The molecular basis and the genes involved in this early decline in IQ during development are unknown. This low IQ corresponds to overall mental retardation. Many studies have investigated the development of short-term memory in individuals with DS. These studies are based on the working memory model with a separation of functions for the processing of verbal material (the phonological loop) and visuospatial material. DS children have been shown to be particularly impaired for the phonological loop component[39,40]. Long-term memory is also impaired in DS patients, who learn visual-spatial sequences normally, but display impairment in the learning of visual-object patterns[41]. Changes in these and other cognitive processes have not yet been linked to the neuropathological features of DS or to a specific phase of development.

In terms of gross morphology, the brains of DS patients are smaller than those of normal subjects. As early as 1966, Crome and Erdohazi reported that the brains of most children with T21 weighed less than those of normal children (76% normal weight) and that low brain stem and cerebellum weights (66% normal weight) were a specific feature of DS[7]. Kemper, in an analysis of measurements taken in six previous studies, showed that the brains of DS patients weighed 24% less than the brains of normal subjects[42]. Only a small number of measurements at birth have been reported, and some studies have indicated that differences in brain size between DS patients and normal subjects may become larger during postnatal brain growth. Very few data have been collected concerning the brain weight of fetuses with T21; a recent study[6] on 1277 fetuses reported a significant decrease, by 12–13%, in brain weight/body weight ratio as early as 15–18 weeks into gestation. In 1990, Schmidt-Sidor et al. suggested that abnormalities probably occurred in the last trimester of pregnancy, between 23 and 40 weeks of gestation[43]. Brain volumes in fetuses have not been assessed by MRI. However, in 1994, Jernigan et al. reported that DS patients aged between 10 and 20 years had total brain volumes 17% lower than those of normal subjects[8]. Pinter et al. compared 15 subjects with a mean age of 11 years and found that mean brain volume was 17.6% lower in patients with DS[9]. Differences in brain size therefore seem to become apparent as early as 15 weeks into gestation, and may increase slightly after birth.

**Table 1**
**Developmental Defects Associated with T21**

| Defects | Age# | Techniques | Ref. |
|---|---|---|---|
| Brain alterations | | | |
| smaller brain weight and brain weight/ body weight | 15-38 w | Brain and body weight | [6] |
| smaller brain and smaller cerebellum | 1-14 y, 10-24y, 5-23y | weight, MRI | [7,8,9] |
| smaller cerebellar diameter | 15-25 w | MRI, ultrasound | [10,11] |
| relatively larger subcortical gray matter | 5-23 y | MRI | [9] |
| smaller hippocampus | 11y | MRI | [9] |
| relative increase of the infratentorial brain region | 15-38 w | weight | [6] |
| decreased frontothalamic distance | 14-21 w | ultrasonography | [11,12] |
| Brain cellular and ultrastructural alterations | | | |
| reduced inferior olivary neuron number | 0,6-28 m | stereology | [13] |
| decrease neuron numbers | 12-19 y | histology | [14] |
| increase nuclear volume | 12-19 y | histology | [14] |
| larger cortical radial cell column | 6 y | histology | [15] |
| altered lamination | 23-32 w | stereology | [16] |
| altered cell layer definition | 14-22 w | histology (Golgi) | [17] |
| larger dendritic arborisation | 0-6 m | Golgi | [17] |
| smaller dendritic arborisation | >2 y, 7m- 11y | Golgi | [17,18] |
| Cranio facial alterations | | | |
| larger brain parietal diameter | 15-35w, 6m-61y, 0,1m-18y | Histology anthropometry | [6,19,20, 21] |
| larger sagittal cerebellar field | 13-26 w | radiography | [22] |
| enlarged cisterna magna | 13-26 w | radiography | [22] |
| cleft palate | infants | | [23] |
| underdevelopment of the maxilla | 6m-61y | anthropometry | [19] |
| nasal bones agenesis | 12-24 w | radiography | [24] |
| Other skeletal and joint alterations | | | |
| ossification of phalanx | 15-40 w | | [21] |
| craniovertebral instability | | radiography | [25] |
| cervical spine malformations | 12-24 w | radiography | [24] |
| iliac angle | 16-17 w | computed tomography | [26] |
| Cardiac anomalies** | | | |
| atrioventricular septal defect & ventricular septal defect | 11-14 w,11-16 w,12-15 w, 13-32 w | Ultrasonography, histology | [27, 28,29,30] |
| aberrant subclavian artery | 18-23 w | ultrasonography | [31] |
| tricuspid regurgitation | 11- 14 w | pulsed Doppler | [32,33] |

**Table 1 continued**

| | | | |
|---|---|---|---|
| Gastrointestinal anomalies | | | |
| hirschsprung | 10d-5m, 30d-1y | histology | [34,35] |
| duodenal atresia | 2y, neonates | clinical exam | [36,37] |
| imperforate anus | neonates | clinical exam | [38] |

* Age is defined as the age at observation.

** Most frequently detected cardiac anomalies; see Table 2 for details and frequencies.

However, this morphogenetic alteration is not homogeneous throughout the brain. Studies of the weight of the infratentorial part of the brain (including the brainstem and cerebellum) have shown that this region is less affected by growth restriction than the supratentorial part of the brain, between 15 and 38 weeks of gestation. This results in the ibw/bw (infratentorial brain weight/total brain weight) ratio being higher in the brains of fetuses with DS[6]. Regional MRI analysis[9] has shown similar differences in the brains of DS children, with an increase in the relative volumes of two regions of the brain with respect to total brain volume: parietal lobe gray matter and subcortical gray matter. Regional differences were also reported in a voxel-based MRI study[44]. The cerebrum seems to be smaller in DS fetuses, as shown by the measurement of various parameters. The frontothalamic distance on ultrasound scans[10,11,12] is 5–10% lower than that of normal fetuses and more than 50% of DS fetuses have frontothalamic distances below the 10th percentile. During fetal development, the decrease in cerebellum dimensions (generally transcerebellar diameter) is in the same range as the decrease in size of the cerebrum (i.e., 5–10%). T21 has a stronger negative effect on cerebellum growth in the last part of gestation and after birth, resulting in the cerebellum being as much as 30% smaller in children with DS than in normal subjects[8,9]. The only part of the cerebrum showing a decrease of this magnitude seems to be the hippocampus (27% decrease[9]).

### *Differences in Cellular Patterns and Ultrastructural Organization*

Histological analyses of DS have shown a decrease in neuronal cell packing density[14,45], particularly in layer III. In the hippocampus, Sylvester noted a decrease in the total number of neurons[46]. In 1997, Pine et al.[13] reported smaller numbers of inferior olivary neurons in 10 patients aged 0.36–28 months (DS/C = 0.64) accompanied by an increase in the volume of individual cells (DS/C = 1.22). Zellweger[47] and Kemper[42] reported difficulties in recognizing cortical layers and cytoarchitectonic areas. Wisniewski et al.[48], in a study of the visual cortex of 60 children with DS from birth to the age of 14 years, reported that DS patients had 20–50% fewer neurons than normal children during this period. They suggested that the neurons were rearranged before birth, particularly in layer IV[48]. Golden and Hyman[16] plotted a set of reference curves for control fetuses, reflecting cell density in the cerebral cortex throughout cortical development. They used these curves as a standard for comparisons. They suggested that migration is normal, but that the second phase, after 20–21 weeks and corresponding to lamination, is both delayed and disorganized in patients with T21. The observed pattern of cortical maturation may reflect an abnormality in axonal and dendritic arborization. In 2002, Buxhoeveden et al.[15] analyzed the size and cell density of a functional anatomical unit of the cortex: the minicolumns. They found that DS patients had larger columns with a lower cell density, indicating a reduction of complexity due to abnormal development. Normal children display expanding dendritic arborization during childhood, whereas patients with DS display greater dendritic branching and total dendritic length than controls in the infantile period (up to the age of 6 months), decreasing steadily thereafter, to significantly below normal levels in the juvenile group (over the age of 2 years)[17,18].

### Craniofacial and Other Skeletal Alterations

Craniofacial alterations were first reported in 1951 by Benda et al., who observed malformations of the head in DS patients[49]. Roche et al.[20] characterized brachycephaly from birth to adulthood by plotting the breadth/length of the head as a function of age; for both male and female patients, this index was much higher for DS patients than for normal children, for all age groups. Allanson et al.[19] also reported brachycephaly in a study of 199 individuals with DS. Skull modifications are observed as early as 15 weeks into gestation. In a large group of 15- to 38-week fetuses, Guihard-Costa et al.[6] found that those with DS had a larger biparietal diameter. This difference was conserved if biparietal diameter was expressed with respect to head circumference. Brachycephaly can also be detected by measuring the biparietal diameter/occipitofrontal diameter ratio[21]. DS patients also show modifications to the osseous component of the cerebellar field: Lomholt et al.[22] performed cephalometric measurements on the cranial X rays of 58 DS patients, and showed that the DS cerebellar field was larger in the sagittal dimension and smaller in the vertical dimension than the normal cerebellar field. This increase in the sagittal dimension of the cerebellar field is consistent with the enlarged cisterna magna seen in DS patients[50].

Other facial modifications have also been reported. Anthropometric analyses[19] have shown underdevelopment of the maxilla in comparison to the mandible (also seen on ultrasound scans of fetuses[51]) and shorter than normal ears have also been reported at 11–14 weeks of gestation[53\2]. Another consequence of developmental defects in DS is nuchal translucency, which is used as a screening test for chromosomal abnormalities[53].

T21 also affects other aspects of skeletal and cartilage development, with agenesis of the nasal bones and malformations of the cervical spine, the most frequently observed abnormalities[24]. Brockmeyer[25] summarized observations concerning craniovertebral instability in DS patients. Two factors may account for this instability: abnormal joint anatomy, with the cup-shaped joints replaced by flat joints, and laxity of one or all of the major occipitoatlantal ligaments responsible for craniovertebral stability. Brockmeyer found evidence of atlantoaxial instability in 10–30% of the DS patients studied. CT scans of pelvic bones have also revealed morphological differences; second-trimester DS fetuses have a significantly greater iliac angle than euploid fetuses[26]. Radiographic measurements of the femur and humerus of DS fetuses showed that these bones were significantly shorter than those of normal fetuses[54]. The cochlea in temporal bones of DS patients have also recently been shown to be shorter than those of euploid individuals[55].

### Cardiac Abnormalities

Congenital heart defects (CHD) are present in about 40–60% of DS infants at birth. These abnormalities include in majority atrioventricular septal defect (AVSD) (with or without other CHDs), ventricular septal defect (VSD) (with or without other CHDs), isolated secundum atrial septal defect (ASD), isolated persistent patent ductus arteriosus (PDA), and isolated tetralogy of Fallot (TOF). The percentage of each category varies between different studies due to the type of population studied (Table 2), indicating that genetic background[56] or consanguinity[57] may play a role in the CHD phenotype. The recent analyses of T21 fetal hearts[58,59] showing that the CHD phenotypes may extend to a smaller anomaly such as the linear insertion of atrioventricular valves (LIAVV) suggest that more T21 patients may bear these anomalies. Heart development in humans occurs very early, starting from the third week to the eighth week of development; from the primitive tube that beats at 25 days to the four-chamber heart. Different steps after formation of the primitive heart tube involve looping, cell migration, cell transition, and septation[for review 60,61,62]. Cardiovascular abnormalities may be detected during fetal development and technical development allows us to detect more of these anomalies. During the first trimester, cardial septal defects are observed and are frequently associated with an increased in nuchal translucency thickness[27,28]; narrowing of aortic isthmus is also observed[29]. From 11 weeks of gestation, tricuspid regurgitation is detected by pulsed wave Doppler in the presence or absence of detectable cardiac defect[32,33]. Percentages of cardiac defects detected in fetuses, mostly AVSD and VSD, may vary between studies[28,63]. During the second and third trimester, an aberrant right subclavian artery, which was previously observed after birth[64], may be also visualized as a new cardiac sign[31].

**TABLE 2**
**Cardiac and Vascular Defects in T21**

| type % | | Quero Jimenez et al. [64] | Freeman et al. [65] | Vida et al. [56] | Venugopalan et al. [57] | Fredouille et al. [59] |
|---|---|---|---|---|---|---|
| number of T21 (fetal) | | | | | | 186 |
| number of T21 (after birth) | | 415 | 227 | 182 | 90 | |
| Total cardiac defects % | | 50 | 44 | 54 | 60 | 77.4 |
| atrioventricular septal defect (complete and partial) | AVSD | 52 | 45 | 9.5 | 27.7 | 43.7 |
| ventricular septal defect | VSD | 26 | 35 | 27.6 | 25.9 | 4* |
| 2nd atrial septal defect | ASD | 3* | 8* (26) | 12.7 | 33.3 | |
| tetralogy of Fallot | TOF | 9 | 4* (5) | | 1.9 | 4* |
| double outlet ventricule | DORV | 0.8 | | 0.5 | | |
| patent ductus arteriosus | PDA | 4 | 7 | 28.6 | 9.3 | |
| mitral abnormalities | | 2 | | | | |
| pulmonary valvar stenosis | | | | | 1.9 | |
| aortic valvar stenosis | | 0.3 | | | 3.2 | |
| coarctation aorta | | 1.5 | 1 | | 1.7 | |
| transposition of great arteries | TGA | | | 0.3 | 1.3 | |
| other | | | 9 | 2 | 4.2** - 2.9*** | |
| linear insertion of atrioventricular valves | LIAVV | | | | | 38 |
| anomalous retrooesophageal subclavian artery | ARSA | 11.5 | | | | |

Comparison of four studies on T21 after birth and recent histological studies on fetal T21 showing the difference of incidence of the different CHD categories. Percentage is indicated when noted in the publication, thus absence of percentage may be either absence of the feature or not reported.
*Corresponds to percentage of isolated anomaly only.
**Complex CHD including DORV, valve atresia and hypoplastic ventricule and
***Miscellaneous as categorized by Venugopalan and Agarwal [57].

### Gastrointestinal Abnormalities

DS patients have a higher frequency of gastrointestinal abnormalities than the diploid population. Hirschsprung disease, a functional intestinal obstruction with aganglionosis, occurs 50 times more frequently in DS patients than in diploid subjects. In rectosigmoid aganglionosis with DS, it has been suggested that certain pathomorphological changes occur in the enteric nervous system[34,35]. Duodenal atresia, in which the duodenal lumen is completely obliterated, is 300 times more frequent in DS patients than in the diploid population[36,66]. The third major malformation associated with DS is an anorectal malformation consisting of a low-lying rectal pouch with no urinogenital or perineal fistula, found in 5–7% of the DS population[38,67].

### ALTERATIONS OF EMBRYOGENESIS AND THE DEVELOPMENT OF MURINE MODELS

For several reasons, it was considered desirable to have animal models of T21. There are restrictions on the study of human tissues for practical and ethical reasons. Only a few cells can be studied and no real access to the central nervous system (CNS) is possible. Due to advances in mouse genetics, it was easier to build these models in mice than in rats. It is possible to follow the developmental stages and to collect cells and tissues from different organs in mice. In addition, the mouse genome contains three large chromosome fragments syntenic for the main parts of HSA 21. Completion of the sequencing of HSA21 and of the mouse genome has made it possible to compare DNA sequences from the two species. The first orthologous chromosome region is located on MMU16 and is syntenic to a region of HSA21 carrying 154 genes; the second is located on MMU17 and corresponds to a region of 23 genes on HSA21; the third is located on MMU10 and corresponds to a region of 58 genes on HSA21 (Fig.1).

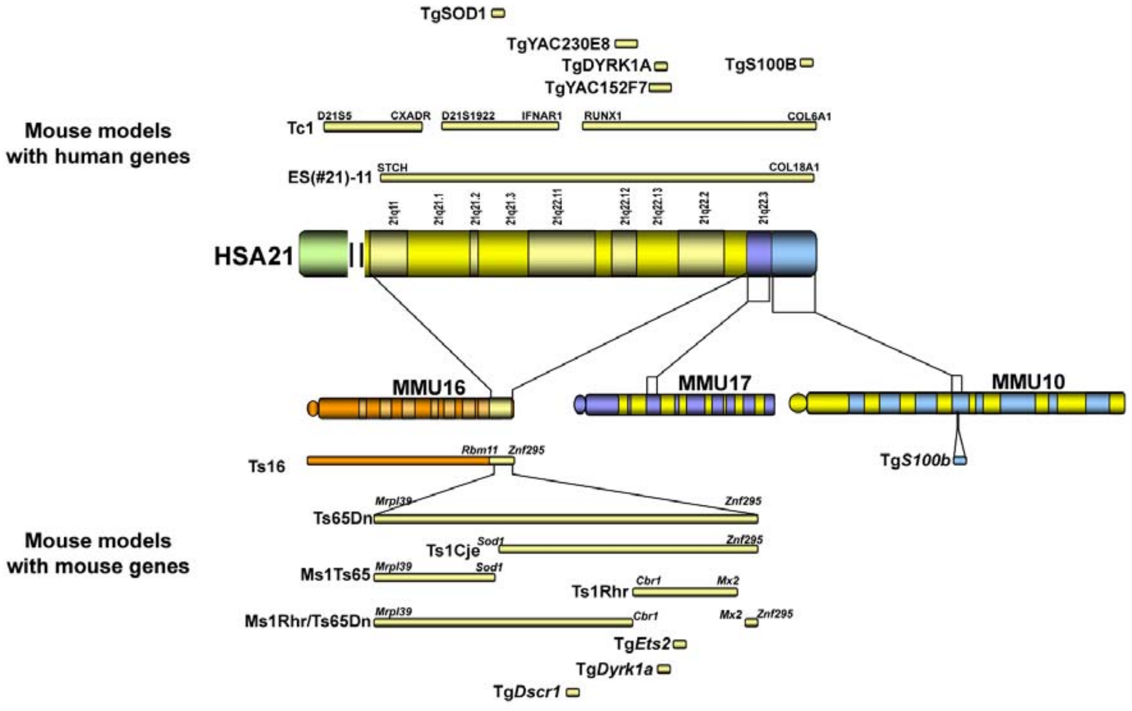

**FIGURE 1.** Mouse models of T21.

### Ts16

The first model to be developed was based on a mouse with 26 chromosomes rather than the usual 40, due to Robertsonian translocations. It was suggested that this model (Ts16 mice) might serve as a valid genetic model for human T21, based on its phenotypic features[68] (Table 3). These features include: (1) a CHD with conotruncal malformations observed in the Ts16 embryos and including a double-outlet right ventricle, persistent truncus arteriosus, tetralogy of Fallot, and right-sided aortic arch[69]; (2) thymic hypoplasia, with the Ts16 thymus containing only 10–20% the number of lymphocytes found in a normal thymus at a comparable stage; (3) a delay in the expression of determinants corresponding to a 2-day lag in the development of fetal trisomic thymocytes[70]; (4) craniofacial abnormalities, including shortened faces, dysplastic ears, and mandibular bone alterations; and (5) midgestational edema, resulting in the thick neck of the fetuses. However, the value of this model is limited by several factors: Ts16 mice die *in utero* and the proximal part of MMU16 contains a large number of genes with human counterparts located on chromosomes other than HSA21 (HSA3, HSA16, HSA22).

**TABLE 3**

**Developmental Defects in Murine Models of T21**

| Trisomy type | Mice | cDNA/gene/segment | Promoter | Additional Copy Number | Phenotype | Ref. |
|---|---|---|---|---|---|---|
| Segmental murine | Ts16 | MMU16: [*Rbm11-Znf295*] (homology with HSA22+HSA3+HSA16) | Gene | 1 | Congenital heart defects; severe thymic hypoplasia; shorter necks; reduced brain weight, midgestational edema | [68] |
| | Ts65Dn | [*Mrpl39-Znf295*] | Gene | 1 | Perinatal loss; cerebellum hypoplasia; craniofacial dysmorphology; brachycephalic skull | [73,74,75] |
| | Ts1Cje | ]*Sod1-Znf295*] | Gene | 1 | Reduced cerebellar volume;craniofacial abnormalities | [78,79] |
| | Ms1Ts65 | [*App-Sod1*[ | Gene | 1 | No developmental abnormalities | [80] |
| | Ts1Rhr | [*Cbr1-Mx2*] | Gene | 1 | Larger mandible | [81] |
| | Ms1Rhr/Ts65Dn | [*Mrpl39-Cbr1*]-[*Mx2-Znf295*] | Gene | 1 | Craniofacial skeleton dysmorphology | [81] |
| Segmental human | ES(#21)-11 | chimeric HSA21 | Gene | 1 | Abnormal body size; thymus hypoplasia; hypoplastic pulmonary artery; double outlet right ventricle; riding aorta; atrioventricular canal hypoplasia | [82] |
| | Tc1 | chimeric HSA21 | Gene | 1 | Lower neuronal density in the cerebellum; perimembranous ventricular septal defect; atrioventricular septal defect | [83] |
| | Tg YAC152F7 | [DSCR5-DYRK1A] | Gene | 1 | Increased brain weight and neuronal size | [8,85] |
| | Tg YAC230E8 | [C21ORF18-CLDN14] | Gene | 1 | increased cortical neuronal density | [84,102] |
| Double gene | Tg *Dyrk1a*-Tg*Dscr1* | Murine cDNA | beta-actin | overexp x2 | failure of heart valve elongation | [88] |

| | Table 3 continued | | | | | |
|---|---|---|---|---|---|---|
| Single gene | Tg SOD1 | Human gene | Gene | 10 | early thymic architecture disorganisation | [103] |
| | BAC DYRK1A | Human gene | Gene | 1 | increased brain weight | [86] |
| | Tg *Dyrk1a* | Murine cDNA | Metalloth ionein | 2-20 | Delayed cranio-caudal maturation | [87] |
| | Tg *Dyrk1a* | Murine cDNA | beta-actin | overexp. x2 | vascular defects, blocked heart valve development | [88] |
| | Tg *Ets2* | Murine cDNA | Metalloth ionein | overexp x2 | Neurocranial, viscerocranial and cervical skeletal abnormalities; thymus abnormalities | [89] |
| | TgS100B | Human gene | Gene | 70 | increased dendritic density in hippocampus ( young mice) | [90,91] |
| | Tg*S100b* | Murine gene | Gene | 2-12 | Alteration in astrocyte morphology and axonal sprouting | [92] |

### Ts65Dn

These limitations led to efforts to construct other segmental trisomies. Davisson et al.[71] used radiation-induced translocations to produce Ts65Dn, a mouse trisomic for a long fragment of MMU16 syntenic to segment MRPL39-ZNF295 in humans. These regions extend on 14 Mb in the mouse and on 16.5 Mb in humans; the human region contains 132 confirmed genes as stated in [72], corresponding to 195 gene IDs and to 154 gene IDs in the mouse (ENSEMBL v40 www.ensembl.org). Large numbers of these mice died in the perinatal period, probably due to developmental defects, reducing the proportion of trisomic mice to 20–40% at weaning, with fewer males (34% of the total progeny) than females (39% of the total progeny)[73]. The males are sterile. Stocks for the translocation are maintained by mating the carriers with a (B6C3H) F1 hybrid. The use of two inbred strains to generate the genetic background may increase phenotype variability. These mice tend to be small and have no reported heart defect. However, in two areas — the CNS and the craniofacial bones — Ts65Dn mice display very interesting abnormalities. MRI showed that the volume of the cerebellum is significantly smaller (12% decrease[74]) in these mice than in normal mice. These results were confirmed by area measurements of histological sections, which also showed a smaller internal granule layer and molecular layer of the cerebellum. Roper et al.[75] showed that trisomic mice have significantly fewer mitotic granule cell precursors than normal mice. In contrast, the brain, with the exception of the cerebellum, is not significantly smaller in segmentally trisomic mice and, indeed, tends to be larger than that of euploid mice if measurements of area at midline level are taken into account (9% increase). Slight variation in hippocampus volume[76] has been observed in the CA2 region. These mice also have significantly fewer neurons in the dentate gyrus and a larger CA3 region than euploid mice. Richtsmeier et al.[77] used three-dimensional morphometric methods to analyze the craniofacial skeleton of Ts65Dn mice. They revealed that linear distances in the rostral part of the face were significantly shorter in these mice. In the neurocranium, only linear distances along the rostrocaudal axis were shorter. By contrast, linear distances along the mediolateral axis in Ts65Dn mice were either similar to or larger than those in euploid mice.

### Ts1Cje/Ms1Ts65

A second partial Ts16 model has recently been developed, the Ts1Cje mouse[78]. This mouse resulted from a reciprocal translocation between the end of chromosome 12 and the distal part of chromosome 16 at the level of the *Sod1* gene; the partial Ts16 resulting from this event contains functional genes distal to *Sod1* (one copy of *Sod1* has been knocked out). The region present in three copies is syntenic to a smaller fragment than the syntenic region in Ts65Dn mice, corresponding to only 85 human genes. Males are fertile. As reported for Ts65Dn mice, Ts1Cje mice have significantly smaller cerebellum volume (12%) and granule cell density than normal mice, although these effects are less marked than in Ts65Dn mice. However, unlike Ts65Dn mice, they display no significant decrease in Purkinje cell density. For most craniofacial measurements, the differences between Ts1Cje mice and their euploid littermates were similar to those between Ts65Dn mice and euploid mice[79]. These results suggest that craniofacial dysmorphology results largely from an imbalance in the genes distal to *Sod1* on MMU16. The Ms1Ts65 mouse, resulting from a cross between mice with a balanced T(16;12)1Cje translocation and Ts65Dn mice, produced a new line trisomic for the fragment present in two copies in Ts1Cje and in three copies in Ts65Dn mice. These mice have normal cerebellar volume and Purkinje cell density[80]. However, they have a significantly lower granule cell density than euploid mice, approximately equivalent to that in Ts1Cje mice, indicating that the larger decrease in granule cell density observed in Ts65Dn mice probably resulted from the triplication of genes from both regions.

### Ts1Rhr/Ms1Rhr

Olson et al.[81] further dissected Ts65Dn mouse phenotypes by constructing a segmental monosomy (Ms1Rhr) and a segmental trisomy (Ts1Rhr), targeting the region between Cbr1 and Mx2 using a Cre-lox strategy. Both lines are fertile. Unlike Ts65Dn mice, Ts1Rhr mice have significantly larger skulls than euploid mice, with overall rostrocaudal elongation; these mice also have larger mandibles (increase in 5 of 21 linear distances). A model with trisomy of the proximal part of the Ts65Dn duplication was built by crossing Ms1Rhr mice with Ts65Dn mice. The resulting mice are trisomic for 70% of the genes trisomic in Ts65Dn and have a smaller skull than their littermates, but with no signs of brachycephaly.

### Transchromosomic Mice Models

Another approach was developed by Shinohara et al.[82], who used microcell-mediated chromosome transfer to generate chimeric mice containing part of human chromosome 21 as an independent chromosome. These chimeric mice had several developmental abnormalities, which tended to be more pronounced in animals with a high HSA21 retention rate. Thymic hypoplasia was frequently observed in fetuses with more than 90% HSA21 retention. These mice also presented cardiovascular abnormalities, with atrioventricular canal hypoplasia observed in mice with an HSA21 retention rate of at least 48%. Conotruncal malformations were observed in fetuses with more than 80% HSA21 retention; these malformations included double-outlet right ventricle and one case of overriding aorta.

O'Doherty et al.[83] used a similar approach. They reproduced human-mouse transchromosomic cell lines on a female background and established the transmission of an almost complete HSA21 of 42 Mb (90%). The resulting colony (Tc1) could be maintained only in a hybrid background (C57BL/6Jx129S8). Differential retention of the human chromosome fragment was observed in the various organs tested, with the proportion of positive cells ranging from 24–66%. Granule neuron density was significantly lower (15–16%) than normal in these mice. No other anatomic or cytoarchitectonic defects were found in Tc1 mice. Tc1 skulls were indistinguishable from wild-type controls in terms of their overall dimensions. Most mandible measurements were also similar, but Tc1 mandibles were significantly smaller between the coronoid process and the mandibular angle, and between the coronoid process and the uppermost point on the incisor alveolar rim. Heart development was analyzed in Tc1 E14.5 embryos; perimembranous ventricular septal defect, corresponding to a lack of fusion between the ventricular septum and the proximal outflow tract cushions, was observed in Tc1 mice. This was associated with an overriding aorta in only one case.

### Other Models

Smith and colleagues[84] used smaller human chromosome fragments inserted into yeast artificial chromosomes to create an *in vivo* library spanning 1.8 Mb of 21q22.2. Two YAC-transgenic mice presented brain abnormalities: tg230E8 (with 9 genes) had a high density of cortical neurons and tg152F7 (with 5 genes including DYRK1A, encoding a serine threonine kinase) had a 15% heavier brain, with larger cortical (layer V) and hippocampal (dentate gyrus) neurons (15%) than euploid mice[85]. A smaller human fragment containing only the DYRK1A gene was used by Ahn et al.[86] to generate a line of transgenic mice with heavier than normal brains (19% heavier).

Other models with overexpression of a single HSA21 gene have been constructed, and some of these models also present several developmental abnormalities. Altafaj et al.[87] generated transgenic mice overexpressing the full-length cDNA of murine *Dyrk1a* under the control of a metallothionein promoter; these mice showed no major structural change in the CNS, but neurodevelopmental analysis suggested that the acquisition and maturation of locomotion from cranial to caudal parts of the body was delayed.

Arron and colleagues[88] constructed a model in which *Dyrk1a* is overexpressed under the control of the actin promoter, resulting in cardiac modifications; two- to threefold overexpression is sufficient to induce vascular defects and block heart valve development.

Sumarsono et al.[89] constructed a transgenic mouse in which the *Ets2* (a transcription factor) cDNA is expressed under the control of a metallothionein promoter. They found that less than twofold overexpression of this cDNA led to the development of neurocranial, viscerocranial, and cervical skeletal abnormalities in these transgenic mice.

A human HSA21 fragment (17 kb), containing the S100beta gene, encoding a calcium-binding protein, was used by Friend et al.[90] for the construction of transgenic mouse lines with a high copy number (10–100) and similarly high levels of protein production. At 5 weeks, these mice presented intense MAP-2 staining in the strata radiatum and stratum lacunosum-moleculare of the CA region and the molecular layer of the dentate gyrus, suggesting a high rate of dendritic maturation[91]. Reeves et al.[92] constructed two lines overexpressing *S100beta*: strong expression of the murine gene resulted in an increase of the radial fibers throughout the granule cells of the dentate gyrus and an increase in immunoreactivity with antibodies directed against axonal proteins, consistent with an increase in axon proliferation.

## COMPARISON OF THE EFFECTS OF INCREASING GENE DOSAGE IN HUMANS AND MICE

### Phenotypes

DS may manifest in the form of phenotypes common to all affected individuals, but of variable intensity, such as mental retardation, and in the form of phenotypes present in only some patients, such as heart defects.

Changes in brain development lead to hypoplasia of various parts of the cerebrum. However, a lack of reliable postmortem or MRI observations makes it impossible to estimate accurately the percentage of patients in which these abnormalities occur. Based on existing series of data, the brain seems to be smaller than normal in 35% of cases, and cerebellum hypoplasia is observed in more than 90% of cases. None of the existing murine models displays a decrease in cerebrum size. Indeed, one model, the Ts65Dn mouse[74], displays a 5% increase in volume and a 9% increase in area measured in the center of the brain, and two models in which human DYRK1A is overexpressed present a 15% increase in brain size. Only two of the existing mouse models — Ts65Dn and Ts1Cje — present hypoplasia of the cerebellum, of up to 12%

in adult animals, much lower than the 25–30% hypoplasia observed on average in patients with T21. The two transchromosomic models with a human HSA21 show no such volume decrease.

Another characteristic of the decreases in brain volume observed in DS patients is their regional heterogeneity, as shown by MRI, and the only model displaying specific volume changes in the equivalent of the subcortical gray matter region is a model in which human DYRK1A is overexpressed[94].

Another characteristic brain phenotype in patients with DS is regional changes in neuron and dendrite densities. No delayed lamination has been found in murine models. By contrast, three models (Ts65Dn, 24%; Ts1Cje, 10%; Tc1, 15%) have low granule cell densities in the cerebellum, mimicking the decrease in granule cell density in DS patients. The dendritic abnormalities early in the patient's life[17] may vary in opposite directions, with an early increase followed by a decrease. Mice transgenic for S100beta have high dendritic densities when young. However, it should be noted that this phenotype is obtained with 70 extra copies.

Comparisons between human and mouse model phenotypes also reveal similarities and discrepancies in terms of skeletal modifications. Not all DS patients display microcephaly, and the main feature observed is brachycephaly. A similar phenotype is observed with Ts65Dn and Ts1Cje mice, but not with mice trisomic for the proximal part of the Ts65Dn segment or with Tc1 mice with 90% of HSA21.

The equivalent of the facial modifications observed in DS patients can be observed in Ts65Dn and Ts1Cje mice, which display decreases in the distances characterizing the frontal part of the skull. No such modifications are found in Tc1 mice or Ts1Rhr mice. Conversely, Ts65Dn, Ts1Cje, and Tc1 mice have smaller than normal mandibles, comparable to the smaller maxilla observed in DS patients, but the decrease in size is greater for the maxilla than for the mandible in humans.

DS fetuses display nuchal translucency. An edema, resulting in a thick neck, is observed in Ts16 mice, but not reported in the other models.

Heart abnormalities are found in the two transchromosomic models — ES (#21)-11 and Tc1 (although at different levels). In the fetuses of both these models, the atrioventicular septal defect responsible for 45% of cardiac abnormalities in DS patients is either absent or rare (1/10 in Tc1); conversely, 50% of Tc1 mice present ventricular septal defect, the second most frequent cardiac abnormality in DS patients. ES (#21)-11 mice have a high frequency of DORV and overriding aorta, reflecting defects in neural crest generation. DORV is rarely observed in DS (Table 2). Furthermore, these mice display a delayed differentiation of cardiomyocytes derived from ES cells carrying this extrachromosomal material.

A third model, in which *Dyrk1a* is overexpressed (from a beta actin promoter) displays certain cardiac modifications that are worsened by the overexpression of this gene together with *Dscr1*, another HSA21 gene ortholog. DSCR1 does not seem to be involved in Tc1 mice phenotype, as it is not present in triplicate in these mice. Thus, while transcription factors like Nkx2-5, GATA4, MEF-2, Tbx family are known to play a role very early in the heart development[94], no gene on chromosome 21 has been definitively confirmed to play a role in the heart defects observed in T21[95]. Moreover, HSA21 genes, orthologs of which in the mouse have been shown to be expressed in the structures of the heart involved in the CHDs[96,97,98,99,100], have been little studied during human heart development[101].

No features comparable to the gastrointestinal abnormalities observed in DS patients have yet been reported in a murine model.

### Molecular Comparisons

The high synteny between distal MMU16 and HSA21 in Ts65Dn and Ts1Cje mice, and the use of human genomic fragments in chimeric transchromosomic models, may account for the similarity in developmental abnormalities observed between these models and humans with DS. However, there are also differences between mouse models and human trisomy and between mouse models with the same targeted genes.

The hypothesis that gene action in DS is qualitative or gene dosage dependent predicts that a region critical for specific phenotypes contains a dosage-sensitive gene or genes, for which a dosage imbalance is sufficient to cause these phenotypes[37,104,105]. An alternative hypothesis, the developmental instability hypothesis, asserts that DS phenotypes result from small nonspecific effects of hundreds of genes with a dosage imbalance, and therefore predicts that all large segments should have the same consequences, regardless of which genes are present in triplicate[106,109]. Long-term observations of human patients with various chromosomal aneuploidies are not consistent with this second hypothesis, as they have shown that the phenotype depends on the set of genes involved and not on the size of the fragment. Similar findings have been reported for mice with various types of partial trisomy or with transchromosomic material[82]. According to a third hypothesis, compatible with the qualitative hypothesis, a critical threshold for an HSA 21 gene product or for a gene product encoded by a different chromosome may be associated with the appearance of a specific phenotype. A cascade effect may increase or decrease the expression of an HSA21 gene, causing levels of the corresponding gene product to cross a critical threshold. If a gene is under the control of two genes on HSA21, identical or similar phenotypes will result from the duplication of either of the two regions carrying these genes. A similar effect may also account for the differences observed between humans and mice, if one species is more sensitive than the other to a specific increase. These thresholds clearly depend on genetic background, accounting for the phenotypic variability observed in humans: DS phenotypes are either present or absent (heart defects or cerebellum hypoplasia) or differ between individuals in terms of their severity (mental retardation).

The modeling of T21 in mice using mouse genes is based on the assumption that both species contain the same set of genes. A systematic analysis of homology between human and mouse transcripts[109] identified three classes of transcript: 170 genes are highly conserved and may be considered human/mouse orthologs, 83 are minimally conserved and may be orthologs (these two categories also include 31 antisense transcripts), and a third category of genes were identified as species specific (111 in humans and 38 in mice). The human-specific genes include a group of primate-specific genes. These differences should be considered when comparing phenotypes in the two species.

The second assumption of this approach is that the trisomy of a given gene or a given fragment will produce the same quantitative effects on the products of the corresponding genes in both species. It is therefore of prime importance to collect data on the levels of mRNAs or proteins in various aneuploid situations and in various tissues at different developmental stages.

In Ts65Dn mice, two groups have carried out transcriptome analyses in multiple tissues[109,110]. Lyle et al. assessed 78 genes by quantitative RT-PCR and found that 37% were expressed at theoretical levels of 1.5 times normal levels, 45% were expressed significantly less strongly than 1.5 times normal levels, and 9% were not significantly overexpressed. Kahlem et al. showed that most of the genes present in triplicate were overexpressed, a few showed down-regulation, compensation, or strong tissue-specific overexpression. This tissue specificity is exemplified by the study of Arron et al.[88] who assessed the expression of two genes, *Dyrk1a* and *Dscr1*, identified as potentially responsible for certain DS phenotypes. They observed *Dyrk1a* overexpression in Ts1Cje embryos on E13.5, but found no increase in Dyrk1a or Dscr1 protein levels in the P1 hippocampal neurons of Ts65Dn mice.

In a study of a subset of HSA21 genes in human amniocytes[111], these genes were found to display 1.1-fold overexpression on average. Mao et al.[113] reported that most of the quantified HSA21 genes were overexpressed in human astrocyte cell lines. The same authors later reported that 22 HSA21 genes displayed 1.75-fold overexpression in the heart, brain, and astrocytes of 17- to 20-week-old fetuses. It is difficult to compare the transcriptome analyses performed in humans with the results obtained in mice, as different tissues and different developmental stages were used. From existing reports, it is possible to identify the genes overexpressed when present in triplicate in human and mouse tissues. This list contains only 18 genes (Table 4), more than half of which are located on the proximal part of HSA21, on 21q21. The paucity of genes significantly overexpressed in both species may reflect the lack of models for the partial trisomy of MMU17 or MMU10. It may also reflect the incompleteness of the set of HSA21 genes on commercial microarrays and the variability of expression levels observed in human samples[113].

**TABLE 4**
**HSA21 and Mouse Orthologs Dosage-Sensitive Genes**

| Genes | References |
|---|---|
| MRLP139 | [109,114] |
| APP | [109,110,111] |
| ZNF294 | [109,110,114,115] |
| USP16 | [110,114] |
| CCT8 | [109,110,114,115,116] |
| SOD1 | [109,110,112,114,116] |
| IL10RB | [109,115,116,117] |
| IFNGR2 | [109,114,116,117,118] |
| GART | [109,110,115,116,117] |
| SON | [109,110,114,118] |
| C21ORF5 | [109,110,115] |
| CRYZL1 | [109,110,112] |
| ATP5O | [109,110,112,117,118] |
| DSCR1 | [109,115,117,118], [110] P30 only |
| WDR9 | [109,115,118] |
| HMGN1 | [115,116,118] |
| WRB | [109,110,114] |
| MX1 | [109,110,115] |
| MX2 | [109,110,115,116] |

### FUTURE RESEARCH

DS is a complex disease, with many phenotypic consequences appearing very early in development. The modeling of DS in mice is particularly complex, due to the size of the region involved and the number of candidate genes to be considered. Gene dosage errors in humans and mouse models are very similar, but there are also clear differences. Increasing our understanding of the pathogenesis of the phenotypic manifestations of trisomy in both species is very important, both to unravel the molecular bases of these defects and for the design of future therapeutic interventions.

The first task in this process is annotation of the gene contents and the transcribed sequence content of mouse and human genomic regions with the same level of accuracy for all types of expressed sequence, including miRNAs, antisense RNAs, and CNGs[72,108,119]. This annotation should also include the tissue specificity of sequence expression during development, and such analyses should be carried out for both species.

Additional mouse models are required. Mouse models with trisomy of all HSA21 syntenic regions (i.e., the MMU segments of chromosomes 16, 17, and 10) should be created and their phenotypes characterized in detail. Mice trisomic for single genes should also be generated to provide information about candidate genes and to obtain results biologically relevant to DS. The transgene (either the mouse gene or the human gene) should be regulated by its own regulatory elements and only one copy should be expressed. In some cases, two or three "single gene" models could be combined[88]. Alternatively, a "gene rescue" approach could be use in which partial trisomic mice are crossed with mice heterozygous for the invalidation of a candidate gene for a given phenotype[120]. This strategy failed to correct the heart defect of Ts16 mice following the crossing of these mice with *Dscr1* (+/-) mice[121]. This last strategy could also be useful for the rapid dissection of important phenotypes, if used to combine large regions of trisomy with smaller monosomic regions. As DS phenotypes are variable, it would also be particularly useful to identify mouse genetic backgrounds in which a given phenotype will be present or absent, to mimic the situation in humans.

An increase in gene dosage may increase protein levels by the expected factor of 1.5, by a factor significantly lower or higher than 1.5, or may result in levels similar to those observed in the euploid state[110]. Some of the increased genes may have trans-acting effects; such effects have been revealed by global transcriptome studies[111,114,116,117,122]. These analyses should be further extended in human and mouse tissues at different developmental stages. The variability of these effects may parallel phenotypic variability.

The results of such analyses may lead to initial therapeutic assays, using pharmacological agents, antibody or antisense technologies, to evaluate the reversal or prevention of some of the effects of T21. Murine models of the overexpression of HSA21 genes involved in DS are likely to be useful for this purpose because they should make possible evaluation of the effects of drugs, antisense molecules, or antibodies in the reversion or prevention of specific modifications in DS patients.
